# The Subtleties and Contrasts of the LeuO Regulator in *Salmonella* Typhi: Implications in the Immune Response

**DOI:** 10.3389/fimmu.2014.00581

**Published:** 2014-12-12

**Authors:** Carmen Guadarrama, Tomás Villaseñor, Edmundo Calva

**Affiliations:** ^1^Departamento de Microbiología Molecular, Instituto de Biotecnología, Universidad Nacional Autónoma de México, Cuernavaca, Mexico; ^2^Departamento de Medicina Molecular y Bioprocesos, Instituto de Biotecnología, Universidad Nacional Autónoma de México, Cuernavaca, Mexico

**Keywords:** LeuO, Typhi, OmpS1, OmpS2, H-NS, porins

## Abstract

*Salmonella* are facultative intracellular pathogens. *Salmonella* infection occurs mainly by expression of two *Salmonella* pathogenicity Islands (SPI-1 and SPI-2). SPI-1 encodes transcriptional factors that participate in the expression of virulence factors encoded in the island. However, there are transcriptional factors encoded outside the island that also participate in the expression of SPI-1-encoded genes. Upon infection, bacteria are capable of avoiding the host immune response with several strategies that involve several virulence factors under the control of transcriptional regulators. Interestingly, LeuO a transcriptional global regulator which is encoded outside of any SPI, is proposed to be part of a complex regulatory network that involves expression of several genes that help bacteria to survive stress conditions and, also, induces the expression of porins that have been shown to be immunogens and can thus be considered as antigenic candidates for acellular vaccines. Hence, the understanding of the LeuO regulon implies a role of bacterial genetic regulation in determining the host immune response.

## Introduction

*Salmonella enterica* are Gram-negative bacterial pathogens capable of infecting human beings and other vertebrates, and causing substantial morbidity and mortality (1, 2). In human beings, most of *Salmonella* serovars can cause infections in the small intestine and hence gastroenteritis; yet a small percentage of *Salmonella* serovars can cause a systemic infection, such as typhoid fever by the Typhi serovar (3). Control of *Salmonella* infection is difficult, in part due to the capacity of the bacterium to tolerate environmental stress, to its widespread distribution, multiple drug resistance, and adaptability (4). They infect human beings and other animals by the fecal–oral route, via contaminated food and water.

After oral acquisition, *Salmonella* resists low pH in the stomach and colonizes the intestinal tract and some cells can disseminate to cause systemic infection of organs such as liver and spleen (1). *Salmonella* virulence factors as well as host immune responses are determinant in the infectious process developed in the pathology (5). *S. enterica* Typhimurium and Typhi serovars interact with host cells through the activities mainly of two type three secretion systems (TTSS), encoded in two pathogenicity islands, 1 and 2 (SPI-1 and SPI-2) (6, 7). While SPI-1 participates in bacterial cell entry into non-phagocytic epithelial cells, SPI-2 is required for intracellular maintenance of the bacteria in a specialized membranous compartment (8). *Salmonella* internalization is mediated by effectors encoded in SPI-1: SopE, SopE2, and SopB, which activate the Rho family of GTPases Rac1, Cdc42 and RhoG (9, 10). These bacterial effectors promote a transcriptional reprograming in host cells, which in turn leads to the expression of pro-inflammatory cytokines, which could be essential for the initiation of diarrhea, a hallmark of acute *Salmonella* infection. Recently, it has been observed that the expression of the pro-inflammatory cytokine interleukin 22 (IL-22) can be exploited by pathogens, such as *Salmonella*, to suppress the growth of their closest competitors thereby enhancing pathogen colonization of mucosal surfaces (11–13).

Upon infection of intestinal epithelial cells, early transcriptional host responses occur characteristically after the stimulation of the innate immune receptors (14). However, the *Salmonella*-induced responses are unique in that this pathogen is capable of stimulating them independently of innate immune receptors (12), which are largely inactive in the intestinal epithelial cells due to robust negative regulatory mechanisms (15–17). After internalization in epithelial cells, bacteria traverse the intestinal epithelium and can invade M-cells overlying Peyer’s patches, as well as being captured by dendritic cells directly from the intestinal lumen (18).

Systemic infection requires intracellular survival and replication, while *Salmonella*-macrophage interactions are essential for bacterial virulence, disease, pathology and chronic infection (19–21). Immunity to intra-macrophage pathogens (i.e., *Salmonella*) requires the infected host to generate a robust and sustained CD4 Th1 response (22). *Salmonella* infection of inbred mouse strains induces a robust CD4^+^ T-cell response that is essential toward protective immunity to secondary infection (23–27). *Salmonella* also induces CD8^+^ T-cells and antibody responses that can contribute to the resolution of infection (25, 27, 28). The first study to successfully characterize *Salmonella*-specific CD4^+^ T-cell clones identified the target antigen of these T-cells as an I-Ak epitope within the central hypervariable portion of bacterial flagellin encoded by the FliC gene (29). Subsequently, additional MHC class II epitopes were identified in the same protein and thus flagellin remains the most thoroughly defined target antigen in the *Salmonella* infection model (30, 31). Additional studies have shown that immunization with flagellin provides a modest degree of protective immunity to *Salmonella* infection, usually defined by slightly lower bacterial counts or a delay in time to death after infection. Thus, flagellin is a well-defined target antigen of CD4^+^ T-cells during *Salmonella* infection and this response contributes modestly to protective immunity *in vivo* (32, 33). Among other antigens, the outer membrane proteins (OMPs) are particularly important. In a murine model, the highly abundant OmpC and OmpF porins (34) can induce long-term antibody responses with high bactericidal capacity, and they even confer protection against challenge with *Salmonella* Typhi (35, 36).

## The LeuO Global Regulator is an LTTR

**Figure 1:**
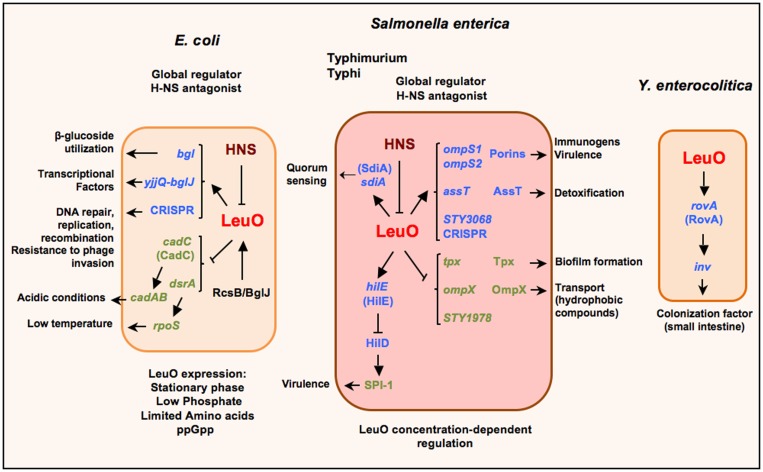
**Schematic representation of the LeuO regulon in *Escherichia coli*, *S*. *enterica* serovars Typhimurium and Typhi, and *Yersinia enterocolitica***. LeuO is a dual regulator that can induce the expression of several genes (arrows) and also is capable of repressing gene expression (lines). When acting as a repressor it has been suggested to function as a backup for H-NS; nevertheless in several cases LeuO acts as a derepressor of gene expression by displacement or prevention of H-NS repression. Recently, LeuO has been denominated as a global antagonist of H-NS in *E. coli* and in *S. enterica* serovar Typhimurium. The expression of *leuO* is repressed by H-NS, although there are some stress conditions when LeuO can be detected in *E. coli*. Also, in *Salmonella* it has been described as an interesting case of differential control of transcriptional regulation, which depends on LeuO concentration. Parentheses depict the proteins coded by the indicated genes. Small arrows denote the several functions for the LeuO-regulated genes.

LeuO is part of the LysR-type transcriptional regulators (LTTRs), the largest family of transcriptional regulators in prokaryotes. In consequence, they regulate a wide variety of genes that are involved in a diversity of cellular functions such as biosynthesis of amino acids, catabolism of aromatic compounds, antibiotic resistance, oxidative stress response, nitrogen fixation, quorum sensing and virulence (Figure 1) (37–40). Many structural studies have shown an organization of an N-terminal DNA-binding domain (DBD) with a winged Helix-Turn-Helix (wHTH) motif; and a long linker helix (LH) involved in dimerization that connects the DBD with the C-terminal effector binding domain (EBD) or regulatory domain (RD) (37, 41–43). These regulators are proteins between 300 and 350 residues, mostly acting as transcriptional activators that bind to A–T rich DNA sequences in similar positions.

In the classical model of action, LTTRs activate the transcription of a divergent gene and repress their own transcription, independently of the presence of a co-inducer or effector (small signal molecule); although there are exceptions where no co-inducer is required and in most of these cases they act as repressors (37). Therefore, the members of the family have been described as dual regulators (44). Nevertheless, there are examples where the LTTR positively autoregulates its expression; and some LTTRs can have more gene targets that they activate or repress, involved in different cellular process, different from those divergently located with respect to the gene for the regulator (39). Even more, as addressed below, LeuO is an interesting case due to the fact that it can act as derepressor, and has been shown to have complex DNA-binding sites (45, 46).

## LeuO History

The first report of the LeuO regulator was by the localization of the *leuO* gene between the *leuABCD* and *ilvIH* operons; upon which it was included in the LysR family due to its amino acid sequence similitude with other members of the family (47, 48). Based on the localization of its gene, LeuO was presumed to be a *leuABCD* regulator, although Leu auxotrophy was not observed in a *leuO* mutant strain (49).

Nevertheless, since the first report of LeuO as a transcriptional regulator, it was shown to be involved in the regulation of genes important for bacterial survival in stringent conditions (Figure 1). Thereby, when LeuO was overexpressed in *E. coli* it was found to repress *cadC*: this was the result of searching for genes that can complement an H-NS mutant strain, thus providing an insight about a relationship between LeuO and H-NS (50). CadC activates the *cadAB* operon, an important system expressed under acidic conditions (51). H-NS is a global regulator that acts as a nucleoid protein (52, 53). Later, LeuO was determined to reduce *rpoS* translation (which encodes S sigma factor) by repression of the small regulatory DsrA-RNA, who positively regulates *rpoS* translation, mainly at low temperature (54). Both *cadC* and *dsrA* are repressed by H-NS (55, 56). Interestingly, in both cases, LeuO indirectly represses the *cadAB* operon expression and RpoS translation.

According with a LeuO-dual role regulator, it was found to be a positive regulator of *bgl* and *yjjQ*-*bglJ* operons in *E. coli*. Later, it was demonstrated that LeuO counteracts H-NS repression (49, 57, 58). The *bgl* operon is involved in the utilization of some β-glucosides as salicin and arbutirin; and the *yjjQ*-*bglJ* genes encode for a transcriptional factor belonging to the LuxR family. These operons are repressed by H-NS in a wild type genotype (59) (Figure 1).

In several studies in *Salmonella* Typhimurium, a model called cis-acting promoter relay mechanism has been described that involves LeuO and DNA local supercoiling in a complex regulatory interplay, in a strain with a mutated promoter of *leuABCD* (p*leuO*-500), and a suppressor mutation in *topA* (60–62). In this complex regulatory mechanism, the Leucine-responsive regulator protein (Lrp) elicits changes in local DNA supercoiling by *ilvIH* promoter activation, exposing the *leuO* regulatory region upon which *leuO* can be transcribed (63–65). Also, there are H-NS binding sites in the regulatory region of *leuO*: hence the system appears to be repressed by changes in local supercoiling and LeuO prevents a cis-spreading of H-NS enhancing positive autoregulation and permits *leuABCD* transcription (66–69).

## The LeuO Regulator in Other Gram-Negative Bacteria

Studies in *S. enterica* serovar Typhi (Figure 1) have shown that overexpression of LeuO induces the expression of two quiescent genes that encode for the OmpS1 and OmpS2 porins (70, 71). An interesting observation was that the LeuO concentration differentially affects *ompS1* and *ompS2* expression. The *ompS2* gene is expressed at lower concentrations of LeuO, whereas *ompS1* is expressed at higher concentrations where *ompS2* expression is repressed. Moreover, for the first time, in a detailed study of *ompS1* expression, LeuO was shown to exert an antagonist role toward H-NS (71). The relevance of this observation is that such function had not been reported for other LTTRs members until now. Interestingly, members of other transcriptional regulators families such as VirF (AraC/XilS), RovA (SlyA/Hor), and Ler (H-NS/StpA) have been described as antagonists of H-NS mainly on genes involved in virulence (72–74).

In a subsequent study to pursue more targets in *Salmonella* Typhi, LeuO was found to also positively regulate *assT* and STY3070; and negatively *ompX*, *tpx* and STY1978 (Figure 1). These genes are involved in a variety of cellular functions (75). AssT is a putative arylsulfate sulfotransferase that has been proposed to be involved in detoxification by transforming toxic phenolic derivatives into non-toxic compounds (76). The global regulators H-NS and LeuO regulate the *assT-dsbL-dsbI* cluster expression negatively and positively, respectively, and this regulation depends on specific growth conditions (77). STY3070 in *Salmonella* was later determined to be the *casC* gene of the CRISPR/Cas system; and its repression was found to depend also on Lrp, and its expression induced in minimal media independent of LeuO (78).

The CRISPR/Cas system in *Escherichia coli* has been involved in DNA repair, replication and recombination and is proposed to confer resistance to phage invasion in bacteria and archaea, thus the suggestion that it is an ancient defense mechanism (79). Interestingly, LeuO was shown to be an antagonist of H-NS in the CRISPR-system in *E. coli* (80). OmpX is an OMP that is homolog to PagC and Rck and Ail proteins of *Salmonella* and *Yersinia*, respectively. When overexpressed, it has been observed to increase sigma E activity; and the lack of *ompX* increased the tolerance to sodium dodecyl sulfate and antibiotics, thus appearing to affect the transport of hydrophobic compounds across the membrane (81–84). Tpx is a thiol peroxidase that codes for a periplasmic antioxidant enzyme that is induced during the exponential growth phase and during biofilm formation (85). It is important to notice that LeuO down-regulates proteins that are involved in the resistance to different pH conditions (83). Another down-regulated gene was STY1978, which codes for a hypothetical protein without an association to any cellular process until now. In this report, LeuO was denominated as a global regulator and opened the possibility that LeuO could have more targets depending on the growth conditions (75).

In *Y. enterocolitica*, LeuO was found to positively regulate *rovA* and, in turn, H-NS also negatively regulates its expression (86) (Figure 1). RovA is a MarA/SlyA type regulator that regulates *inv* gene expression in response to temperature and growth phase (87).

In *E. coli*, by SELEX screening, LeuO was found to regulate genes involved in sulfa drug sensitivity and to increase its own expression during transition into stationary phase and after a week of culture, where H-NS concentration decreased (Figure 1). Even more, a global antagonistic interplay between H-NS and LeuO was proposed, acting on some genes involved in stress response, such as cryptic chaperone/usher-type fimbriae. In addition, mutants in *leuO* and in some fimbrial genes were defective or altered in biofilm formation (88, 89).

In *S. enterica* serovar Typhimurium, LeuO was reported to increase *sdiA* expression in low levels (90) (Figure 1). SdiA is proposed to respond to signals produced by other organisms (91, 92) and recently was found to be active in gut in response to AHLs (*N*-acyl homoserine lactones) a quorum sensing signal produced by other species (93–96).

In a genomic study in *S. enterica* serovar Typhimurium, using ChIP-chip, the LeuO regulon members were extended to include SPI-1 (Figure 1) and SPI-2 genes. In addition, the differential binding of LeuO and regulation of genes was observed depending on the concentration of LeuO. Another important observation was the intragenic binding; hence opening the possibility that LeuO could act as a negative regulator preventing the progress of transcription or as nucleoid structure protein. The finding of LeuO co-binding at various sites with H-NS and RNA polymerase confirms the notion of the antagonist role of LeuO, although they could likely be acting together to regulate a large number of genes. Moreover, the possible interaction with RNA polymerase and H-NS would suggest another mechanism of LeuO regulation (45, 46).

In this respect, the structural properties of LeuO as an LTTR member have been initially explored: finding that it is active as a tetramer, that the mechanisms for induction and repression of gene expression appear to be different, and that there are relevant interactions between the N- and C-termini (97).

## LeuO Expression Conditions

In the *Salmonella* Typhi and *E. coli* wild type genomic backgrounds, LeuO expression is silenced by H-NS (unpublished data). Nevertheless, in *E. coli* and *Salmonella* Typhimurium, *leuO* expression has been detected when grown under stress conditions, especially in the stationary phase under nutrient limitation. Nevertheless, *leuO* is not under the control of *rpoS*; although its expression requires the presence of ppGpp in stationary phase (54, 63, 98, 99). Interestingly, LeuO was shown to be essential to restore cellular growth, after a 2-h delay in a media lacking isoleucine, valine, and leucine (100).

Also, LeuO expression was detected in a phosphate-restricted media (98); and recently it was shown that the expression of the *leuO* gene can be activated by the RcsB and BglJ regulators (58, 101)

## LeuO has Several Functions *In vivo*

Even though LeuO is expressed at very low level in standard laboratory conditions, it seems that *in vivo* it has a role in bacterial survival. In this manner, in a mouse and in a *Caenorhabditis elegans* model of infection, a *S. enterica* serovar Typhimurium *leuO* mutant showed to be attenuated in virulence. Also, in *Vibrio cholera*, biofilm formation was reduced in a deleted *leuO* strain (102–104).

Virulence attenuation in a murine model was reported for the *ompC ompF* double mutant (105). In addition, it has been observed that the OmpC and OmpF porins induced long-term antibody response with bactericidal capacity and conferred protection against challenge with *Salmonella* Typhi (35, 36). Nevertheless, these major porins are expressed at very high levels in standard laboratory conditions. In addition, strains lacking *ompS1* and *ompS2* are attenuated for virulence, suggesting that besides lacking the LeuO regulator the absence of OmpS1 and OmpS2 porins affected bacterial survival (103). Virulence attenuation of mutated strains in *leuO* and *ompS1* and *ompS2* quiescent genes offers evidence that they are expressed *in vivo*. Even though the specific role of these porins in *Salmonella* virulence is not clear, it has been shown that the major porins are passive diffusion channels of solutes, nutrients and toxins through the outer bacterial membrane that might allow bacteria to grow in different environments and to be resistant to drugs (106).

Recently it was found that OmpS1 and OmpS2 induced a strong immune response in the mouse, and a single dose conferred a significant protection against *Salmonella* Typhi. The immunostimulatory properties of OmpS1 and OmpS2 porins further reinforce the notion that they could be expressed following host infection. These studies are relevant because they open the possibility of using these porins as antigens for the development of vaccines against typhoid fever and other non-typhoidal salmonellosis (107).

Moreover, in a recent report it was shown that the activation of *leuO* transcription in *S. enterica* serovar Typhimurium represses expression of pathogenicity island 1 (SPI-1) and inhibits invasion of epithelial cells (108). Two different modes of action were found: the major one that involves the induction of *hilE* transcription by LeuO (Figure 1) and another one that was HilE-independent. HilE is a regulator encoded outside SPI-1 that represses *hilD* expression. HilD is one of the transcriptional factors encoded in SPI-1 that positively controls the expression of other genes in the island (109, 110). It has been suggested that LeuO repression of SPI-1 genes may occur under growth conditions where H-NS, for unknown reasons, has failed to perform such repression.

The possibility of LeuO acting as a backup for H-NS has two implications: one is that it could allow *Salmonella* to confront the hostile free-living conditions where SPI-1 gene expression has a high cost in bacterial growth; and two, it might ensure the specific, sequential, and appropriate level of SPI-1 gene expression in the intestine (111, 112). Due to the fact that H-NS in *Salmonella* is considered as a genome sentinel that silences horizontally acquired genes (113–115), LeuO could be acting as a backup regulator for H-NS, highlighting the subtleties and contrasts of the LeuO mode of action. Thus, the proposed role of LeuO as an activator or as a repressor depending on its concentration could explain this differential gene regulation.

LeuO is an example of a global regulator whose level of expression is an important issue, since this has an effect on its many regulated genes that are involved in a variety of cellular functions, such as virulence and bacterial survival. The levels of expression could thus have spatial and temporal consequences as well. In addition, knowledge of LeuO-regulated genes has been important in the study of the immune response induced by *Salmonella*, such as that elicited by the quiescent porins, which are protein components of the outer membrane. This has opened the possibility for the development of typhoid fever vaccines and perhaps as adjuvants for others vaccines.

It is intriguing that conditions known at present for LeuO expression are extreme and that in many studies it has to be overexpressed to analyze its function. Furthermore, no co-inducer of LeuO is known until now. These are some of the subjects that pose challenges for the future.

## Conflict of Interest Statement

The authors declare that the research was conducted in the absence of any commercial or financial relationships that could be construed as a potential conflict of interest.

## References

[1] Coburn B Grassl GA Finlay BB. *Salmonella*, the host and disease: a brief review. Immunol Cell Biol (2007) 85(2):112–8. 10.1038/sj.icb.7100007 17146467

[2] Yue M Schifferli DM. Allelic variation in *Salmonella*: an underappreciated driver of adaptation and virulence. Front Microbiol (2014) 4:419. 10.3389/fmicb.2013.00419 24454310 PMC3882659

[3] Mittrucker HW Kaufmann SH. Immune response to infection with *Salmonella typhimurium* in mice. J Leukoc Biol (2000) 67(4):457–63. 10770276 10.1002/jlb.67.4.457

[4] Chen HM Wang Y Su LH Chiu CH. Nontyphoid *Salmonella* infection: microbiology, clinical features, and antimicrobial therapy. Pediatr Neonatol (2013) 54(3):147–52. 10.1016/j.pedneo.2013.01.010 23597525

[5] de Jong HK Parry CM van der Poll T Wiersinga WJ. Host-pathogen interaction in invasive salmonellosis. PLoS Pathog (2012) 8(10):e1002933. 10.1371/journal.ppat.1002933 23055923 PMC3464234

[6] Galan JE. *Salmonella* interactions with host cells: type III secretion at work. Annu Rev Cell Dev Biol (2001) 17:53–86. 10.1146/annurev.cellbio.17.1.53 11687484

[7] Waterman SR Holden DW. Functions and effectors of the *Salmonella* pathogenicity island 2 type III secretion system. Cell Microbiol (2003) 5(8):501–11. 10.1046/j.1462-5822.2003.00294.x 12864810

[8] Hannemann S Gao B Galán JE. *Salmonella* modulation of host cell gene expression promotes its intracellular growth. PLoS Pathog (2013) 9(10):e1003668. 10.1371/journal.ppat.1003668 24098123 PMC3789771

[9] Patel JC Galán JE. Differential activation and function of Rho GTPases during *Salmonella*-host cell interactions. J Cell Biol (2006) 175(3):453–63. 10.1083/jcb.200605144 17074883 PMC2064522

[10] Patel JC Galán JE. Manipulation of the host actin cytoskeleton by *Salmonella* – all in the name of entry. Curr Opin Microbiol (2005) 8(1):10–5. 10.1016/j.mib.2004.09.001 15694851

[11] Hobbie S Chen LM Davis RJ Galán JE. Involvement of mitogen-activated protein kinase pathways in the nuclear responses and cytokine production induced by *Salmonella typhimurium* in cultured intestinal epithelial cells. J Immunol (1997) 159(11):5550–9. 9548496

[12] Bruno VM Hannemann S Lara-Tejero M Flavell RA Kleinstein SH Galán JE. *Salmonella typhimurium* type III secretion effectors stimulate innate immune responses in cultured epithelial cells. PLoS Pathog (2009) 5(8):e1000538. 10.1371/journal.ppat.1000538 19662166 PMC2714975

[13] Behnsen J Jellbauer S Wong CP Edwards RA George MD Ouyang W The cytokine IL-22 promotes pathogen colonization by suppressing related commensal bacteria. Immunity (2014) 40(2):262–73. 10.1016/j.immuni.2014.01.003 24508234 PMC3964146

[14] Jenner RG Young RA. Insights into host responses against pathogens from transcriptional profiling. Nat Rev Microbiol (2005) 3(4):281–94. 10.1038/nrmicro1126 15806094

[15] Lee J Mo JH Shen C Rucker AN Raz E. Toll-like receptor signaling in intestinal epithelial cells contributes to colonic homoeostasis. Curr Opin Gastroenterol (2007) 23(1):27–31. 10.1097/MOG.0b013e3280118272 17133081

[16] Shibolet O Podolsky DK. TLRs in the Gut. IV. Negative regulation of toll-like receptors and intestinal homeostasis: addition by subtraction. Am J Physiol Gastrointest Liver Physiol (2007) 292(6):G1469–73. 10.1152/ajpgi.00531.2006 17554134

[17] Lang T Mansell A. The negative regulation of toll-like receptor and associated pathways. Immunol Cell Biol (2007) 85(6):425–34. 10.1038/sj.icb.7100094 17621314

[18] Fink SL Cookson BT. Pyroptosis and host cell death responses during *Salmonella* infection. Cell Microbiol (2007) 9(11):2562–70. 10.1111/j.1462-5822.2007.01036.x 17714514

[19] Lindgren SW Stojiljkovic I Heffron F. Macrophage killing is an essential virulence mechanism of *Salmonella typhimurium*. Proc Natl Acad Sci U S A (1996) 93(9):4197–201. 10.1073/pnas.93.9.4197 8633040 PMC39511

[20] Wijburg OL Simmons CP van Rooijen N Strugnell RA. Dual role for macrophages in vivo in pathogenesis and control of murine *Salmonella enterica* serovar Typhimurium infections. Eur J Immunol (2000) 30(3):944–53. 10.1002/1521-4141(200003)30:3<944::AID-IMMU944>3.3.CO;2-T 10741413

[21] Monack DM Bouley DM Falkow S. *Salmonella typhimurium* persists within macrophages in the mesenteric lymph nodes of chronically infected Nramp1+/+ mice and can be reactivated by IFNgamma neutralization. J Exp Med (2004) 199(2):231–41. 10.1084/jem.20031319 14734525 PMC2211772

[22] McSorley SJ. Immunity to intestinal pathogens: lessons learned from *Salmonella*. Immunol Rev (2014) 260(1):168–82. 10.1111/imr.12184 24942689 PMC4066191

[23] Mittrucker HW Kohler A Kaufmann SH. Characterization of the murine T-lymphocyte response to *Salmonella enterica* serovar Typhimurium infection. Infect Immun (2002) 70(1):199–203. 10.1128/IAI.70.1.199-203.2002 11748183 PMC127597

[24] Srinivasan A Foley J McSorley SJ. Massive number of antigen-specific CD4 T cells during vaccination with live attenuated *Salmonella* causes interclonal competition. J Immunol (2004) 172(11):6884–93. 10.4049/jimmunol.172.11.6884 15153507

[25] Nauciel C. Role of CD4+ T cells and T-independent mechanisms in acquired resistance to *Salmonella typhimurium* infection. J Immunol (1990) 145(4):1265–9. 1974274

[26] Mastroeni P Villarreal-Ramos B Hormaeche CE. Role of T cells, TNF alpha and IFN gamma in recall of immunity to oral challenge with virulent *Salmonellae* in mice vaccinated with live attenuated aro-*Salmonella* vaccines. Microb Pathog (1992) 13(6):477–91. 10.1016/0882-4010(92)90014-F 1363824

[27] O’Donnell H Pham OH Li LX Atif SM Lee SJ Ravesloot MM Toll-like receptor and inflammasome signals converge to amplify the innate bactericidal capacity of T helper 1 cells. Immunity (2014) 40(2):213–24. 10.1016/j.immuni.2013.12.013 24508233 PMC3960852

[28] Mastroeni P Villarreal-Ramos B Hormaeche CE. Adoptive transfer of immunity to oral challenge with virulent *Salmonellae* in innately susceptible BALB/c mice requires both immune serum and T cells. Infect Immun (1993) 61(9):3981–4. 8359920 10.1128/iai.61.9.3981-3984.1993 PMC281103

[29] Cookson BT Bevan MJ. Identification of a natural T-cell epitope presented by *Salmonella*-infected macrophages and recognized by T cells from orally immunized mice. J Immunol (1997) 158(9):4310–9. 9126993

[30] Bergman MA Cummings LA Alaniz RC Mayeda L Fellnerova I Cookson BT. CD4+-T-cell responses generated during murine *Salmonella enterica* serovar Typhimurium infection are directed towards multiple epitopes within the natural antigen FliC. Infect Immun (2005) 73(11):7226–35. 10.1128/IAI.73.11.7226-7235.2005 16239517 PMC1273846

[31] Salazar-Gonzalez RM McSorley SJ. *Salmonella* flagellin, a microbial target of the innate and adaptive immune system. Immunol Lett (2005) 101(2):117–22. 10.1016/j.imlet.2005.05.004 15975666

[32] McSorley SJ Cookson BT Jenkins MK. Characterization of CD4+ T cell responses during natural infection with *Salmonella typhimurium*. J Immunol (2000) 164(2):986–93. 10.4049/jimmunol.164.2.986 10623848

[33] Strindelius L Degling Wikingsson L Sjoholm I. Extracellular antigens from *Salmonella enteritidis* induce effective immune response in mice after oral vaccination. Infect Immun (2002) 70(3):1434–42. 10.1128/IAI.70.3.1434-1442.2002 11854230 PMC127788

[34] Blanco F Isibasi A Raul Gonzalez C Ortiz V Paniagua J Arreguin C Human cell mediated immunity to porins from *Salmonella typh*i. Scand J Infect Dis (1993) 25(1):73–80. 10.1080/00365549309169673 8384733

[35] Isibasi A Ortiz-Navarrete V Paniagua J Pelayo R Gonzalez CR García JA Active protection of mice against *Salmonella typhi* by immunization with strain-specific porins. Vaccine (1992) 10(12):811–3. 10.1016/0264-410X(92)90041-H 1333686

[36] Secundino I López-Macias C Cervantes-Barragán L Gil-Cruz C Ríos-Sarabia N Pastelín-Palacios R *Salmonella* porins induce a sustained, lifelong specific bactericidal antibody memory response. Immunology (2006) 117(1):59–70. 10.1111/j.1365-2567.2005.02263.x 16423041 PMC1782194

[37] Schell MA. Molecular biology of the LysR family of transcriptional regulators. Annu Rev Microbiol (1993) 47:597–626. 10.1146/annurev.mi.47.100193.003121 8257110

[38] Tropel D van der Meer JR. Bacterial transcriptional regulators for degradation pathways of aromatic compounds. Microbiol Mol Biol Rev (2004) 68(3):474–500. 10.1128/MMBR.68.3.474-500.2004 15353566 PMC515250

[39] Maddocks SE Oyston PC. Structure and function of the LysR-type transcriptional regulator (LTTR) family proteins. Microbiology (2008) 154(Pt 12):3609–23. 10.1099/mic.0.2008/022772-0 19047729

[40] Pareja E Pareja-Tobes P Manrique M Pareja-Tobes E Bonal J Tobes R. Extratrain: a database of extragenic regions and transcriptional information in prokaryotic organisms. BMC Microbiol (2006) 6:29. 10.1186/1471-2180-6-29 16539733 PMC1453763

[41] Muraoka S Okumura R Ogawa N Nonaka T Miyashita K Senda T. Crystal structure of a full-length LysR-type transcriptional regulator, CbnR: unusual combination of two subunit forms and molecular bases for causing and changing DNA bend. J Mol Biol (2003) 328(3):555–66. 10.1016/S0022-2836(03)00312-7 12706716

[42] Sainsbury S Lane LA Ren J Gilbert RJ Saunders NJ Robinson CV The structure of CrgA from *Neisseria meningitidis* reveals a new octameric assembly state for LysR transcriptional regulators. Nucleic Acids Res (2009) 37(14):4545–58. 10.1093/nar/gkp445 19474343 PMC2724274

[43] Zhou X Lou Z Fu S Yang A Shen H Li Z Crystal structure of ArgP from *Mycobacterium tuberculosis* confirms two distinct conformations of full-length LysR transcriptional regulators and reveals its function in DNA binding and transcriptional regulation. J Mol Biol (2010) 396(4):1012–24. 10.1016/j.jmb.2009.12.033 20036253

[44] Perez-Rueda E Collado-Vides J. The repertoire of DNA-binding transcriptional regulators in *Escherichia coli* K-12. Nucleic Acids Res (2000) 28(8):1838–47. 10.1093/nar/28.8.1838 10734204 PMC102813

[45] Dillon SC Espinosa E Hokamp K Ussery DW Casadesús J Dorman CJ. LeuO is a global regulator of gene expression in *Salmonella enterica* serovar Typhimurium. Mol Microbiol (2012) 85(6):1072–89. 10.1111/j.1365-2958.2012.08162.x 22804842

[46] Hernández-Lucas I Calva E. The coming of age of the LeuO regulator. Mol Microbiol (2012) 85(6):1026–8. 10.1111/j.1365-2958.2012.08175.x 22812455

[47] Hertzberg KM Gemmill R Jones J Calvo JM. Cloning of an *Eco*RI-generated fragment of the leucine operon of *Salmonella typhimurium*. Gene (1980) 8(2):135–52. 10.1016/0378-1119(80)90033-5 6987127

[48] Henikoff S Haughn GW Calvo JM Wallace JC. A large family of bacterial activator proteins. Proc Natl Acad Sci U S A (1988) 85(18):6602–6. 10.1073/pnas.85.18.6602 3413113 PMC282025

[49] Ueguchi C Ohta T Seto C Suzuki T Mizuno T. The LeuO gene product has a latent ability to relieve *bgl* silencing in *Escherichia coli*. J Bacteriol (1998) 180(1):190–3. 9422614 10.1128/jb.180.1.190-193.1998 PMC106870

[50] Shi X Bennett GN. Effects of multicopy LeuO on the expression of the acid-inducible lysine decarboxylase gene in *Escherichia coli*. J Bacteriol (1995) 177(3):810–4. 7836317 10.1128/jb.177.3.810-814.1995 PMC176661

[51] Soksawatmaekhin W Kuraishi A Sakata K Kashiwagi K Igarashi K. Excretion and uptake of cadaverine by CadB and its physiological functions in *Escherichia coli*. Mol Microbiol (2004) 51(5):1401–12. 10.1046/j.1365-2958.2003.03913.x 14982633

[52] Rimsky S Spassky A. Sequence determinants for H1 binding on *Escherichia coli lac* and *gal* promoters. Biochemistry (1990) 29(15):3765–71. 10.1021/bi00467a024 2160266

[53] Durrenberger M La Teana A Citro G Venanzi F Gualerzi CO Pon CL. *Escherichia coli* DNA-binding protein H-NS is localized in the nucleoid. Res Microbiol (1991) 142(4):373–80. 10.1016/0923-2508(91)90106-K 1871423

[54] Klauck E Bohringer J Hengge-Aronis R. The LysR-like regulator LeuO in *Escherichia coli* is involved in the translational regulation of *rpoS* by affecting the expression of the small regulatory DsrA-RNA. Mol Microbiol (1997) 25(3):559–69. 10.1046/j.1365-2958.1997.4911852.x 9302018

[55] Shi X Waasdorp BC Bennett GN. Modulation of acid-induced amino acid decarboxylase gene expression by HNS in *Escherichia coli*. J Bacteriol (1993) 175(4):1182–6. 8381784 10.1128/jb.175.4.1182-1186.1993 PMC193036

[56] Yamashino T Ueguchi C Mizuno T. Quantitative control of the stationary phase-specific sigma factor, sigma S, *in Escherichia coli*: involvement of the nucleoid protein H-NS. EMBO J (1995) 14(3):594–602. 7859747 10.1002/j.1460-2075.1995.tb07035.x PMC398118

[57] Bartowsky E Normark S. Purification and mutant analysis of *Citrobacter freundii* AmpR, the regulator for chromosomal AmpC beta-lactamase. Mol Microbiol (1991) 5(7):1715–25. 10.1111/j.1365-2958.1991.tb01920.x 1943705

[58] Stratmann T Madhusudan S Schnetz K. Regulation of the *yjjQ-bglJ* operon, encoding LuxR-type transcription factors, and the divergent *yjjP* gene by H-NS and LeuO. J Bacteriol (2008) 190(3):926–35. 10.1128/JB.01447-07 18055596 PMC2223560

[59] Bertin P Lejeune P Laurent-Winter C Danchin A. Mutations in *bglY*, the structural gene for the DNA-binding protein H1, affect expression of several *Escherichia coli* genes. Biochimie (1990) 72(12):889–91. 10.1016/0300-9084(90)90008-5 2128918

[60] Lilley DM Higgins CF. Local DNA topology and gene expression: the case of the *leu-500* promoter. Mol Microbiol (1991) 5(4):779–83. 10.1111/j.1365-2958.1991.tb00749.x 1857204

[61] Chen D Bowater R Dorman CJ Lilley DM. Activity of a plasmid-borne *leu-500* promoter depends on the transcription and translation of an adjacent gene. Proc Natl Acad Sci U S A (1992) 89(18):8784–8. 10.1073/pnas.89.18.8784 1326763 PMC50005

[62] Wu HY Tan J Fang M. Long-range interaction between two promoters: activation of the *leu-500* promoter by a distant upstream promoter. Cell (1995) 82(3):445–51. 10.1016/0092-8674(95)90433-6 7634334

[63] Fang M Wu HY. Suppression of *leu-500* mutation in *topA*+ *Salmonella typhimurium* strains. The promoter relay at work. J Biol Chem (1998) 273(45):29929–34. 10.1074/jbc.273.45.29929 9792711

[64] Fang M Wu HY. A promoter relay mechanism for sequential gene activation. J Bacteriol (1998) 180(3):626–33. 9457867 10.1128/jb.180.3.626-633.1998 PMC106931

[65] Wu HY Fang M. DNA supercoiling and transcription control: a model from the study of suppression of the *leu-500* mutation in *Salmonella typhimurium topA*-strains. Prog Nucleic Acid Res Mol Biol (2003) 73:43–68. 10.1016/S0079-6603(03)01002-X 12882514

[66] Chen CC Fang M Majumder A Wu HY. A 72-base pair AT-rich DNA sequence element functions as a bacterial gene silencer. J Biol Chem (2001) 276(12):9478–85. 10.1074/jbc.M010501200 11121424

[67] Chen CC Ghole M Majumder A Wang Z Chandana S Wu HY. LeuO-mediated transcriptional derepression. J Biol Chem (2003) 278(39):38094–103. 10.1074/jbc.M300461200 12871947

[68] Chen CC Wu HY. LeuO protein delimits the transcriptionally active and repressive domains on the bacterial chromosome. J Biol Chem (2005) 280(15):15111–21. 10.1074/jbc.M414544200 15711009

[69] Chen CC Chou MY Huang CH Majumder A Wu HY. A cis-spreading nucleoprotein filament is responsible for the gene silencing activity found in the promoter relay mechanism. J Biol Chem (2005) 280(6):5101–12. Epub 2004/12/08., 10.1074/jbc.M411840200 15582999

[70] Fernández-Mora M Puente JL Calva E. OmpR and LeuO positively regulate the *Salmonella enterica* serovar Typhi *ompS2* porin gene. J Bacteriol (2004) 186(10):2909–20. 10.1128/JB.186.10.2909-2920.2004 15126450 PMC400630

[71] De la Cruz MA Fernández-Mora M Guadarrama C Flores-Valdez MA Bustamante VH Vázquez A LeuO antagonizes H-NS and StpA-dependent repression in *Salmonella enterica ompS1*. Mol Microbiol (2007) 66(3):727–43. 10.1111/j.1365-2958.2007.05958.x 17908208

[72] Tobe T Yoshikawa M Mizuno T Sasakawa C. Transcriptional control of the invasion regulatory gene *virB* of *Shigella flexneri*: activation by VirF and repression by H-NS. J Bacteriol (1993) 175(19):6142–9. 7691791 10.1128/jb.175.19.6142-6149.1993 PMC206708

[73] Bustamante VH Santana FJ Calva E Puente JL. Transcriptional regulation of type III secretion genes in enteropathogenic *Escherichia coli*: Ler antagonizes H-NS-dependent repression. Mol Microbiol (2001) 39(3):664–78. 10.1046/j.1365-2958.2001.02209.x 11169107

[74] Heroven AK Nagel G Tran HJ Parr S Dersch P. RovA is autoregulated and antagonizes H-NS-mediated silencing of invasin and *rovA* expression in Y*ersinia pseudotuberculosis*. Mol Microbiol (2004) 53(3):871–88. 10.1111/j.1365-2958.2004.04162.x 15255899

[75] Hernández-Lucas I Gallego-Hernández AL Encarnación S Fernández-Mora M Martínez-Batallar AG Salgado H The LysR-type transcriptional regulator LeuO controls expression of several genes in *Salmonella enterica* serovar Typhi. J Bacteriol (2008) 190(5):1658–70. 10.1128/JB.01649-07 18156266 PMC2258680

[76] Kang JW Kwon AR Kim DH Choi EC. Cloning and sequencing of the *astA* gene encoding arylsulfate sulfotransferase from *Salmonella typhimurium*. Biol Pharm Bull (2001) 24(5):570–4. 10.1248/bpb.24.570 11379783

[77] Gallego-Hernández AL Hernández-Lucas I De la Cruz MA Olvera L Morett E Medina-Aparicio L Transcriptional regulation of the *assT*-*dsbL*-*dsbI* gene cluster in *Salmonella enterica* serovar Typhi IMSS-1 depends on LeuO, H-NS, and specific growth conditions. J Bacteriol (2012) 194(9):2254–64. 10.1128/JB.06164-11 22343301 PMC3347046

[78] Medina-Aparicio L Rebollar-Flores JE Gallego-Hernández AL Vázquez A Olvera L Gutiérrez-Rios RM The CRISPR/*Cas* immune system is an operon regulated by LeuO, H-NS, and leucine-responsive regulatory protein in *Salmonella enterica* serovar Typhi. J Bacteriol (2011) 193(10):2396–407. 10.1128/JB.01480-10 21398529 PMC3133143

[79] Barrangou R Fremaux C Deveau H Richards M Boyaval P Moineau S CRISPR provides acquired resistance against viruses in prokaryotes. Science (2007) 315(5819):1709–12 10.1126/science.1138140 17379808

[80] Westra ER Pul U Heidrich N Jore MM Lundgren M Stratmann T H-NS-mediated repression of CRISPR-based immunity in *Escherichia coli* K12 can be relieved by the transcription activator LeuO. Mol Microbiol (2010) 77(6):1380–93. 10.1111/j.1365-2958.2010.07315.x 20659289

[81] Mecsas J Rouviere PE Erickson JW Donohue TJ Gross CA. The activity of sigma E, an *Escherichia coli* heat-inducible sigma-factor, is modulated by expression of outer membrane proteins. Genes Dev (1993) 7(12B):2618–28. 10.1101/gad.7.12b.2618 8276244

[82] Mecsas J Welch R Erickson JW Gross CA. Identification and characterization of an outer membrane protein, OmpX, in *Escherichia coli* that is homologous to a family of outer membrane proteins including Ail of *Yersinia enterocolitica*. J Bacteriol (1995) 177(3):799–804. 7836315 10.1128/jb.177.3.799-804.1995 PMC176659

[83] Stancik LM Stancik DM Schmidt B Barnhart DM Yoncheva YN Slonczewski JL. pH-dependent expression of periplasmic proteins and amino acid catabolism in *Escherichia coli*. J Bacteriol (2002) 184(15):4246–58. 10.1128/JB.184.15.4246-4258.2002 12107143 PMC135203

[84] Otto K Hermansson M. Inactivation of *ompX* causes increased interactions of type 1 fimbriated *Escherichia coli* with abiotic surfaces. J Bacteriol (2004) 186(1):226–34. 10.1128/JB.186.1.226-234.2004 14679242 PMC303450

[85] Kim YH Lee Y Kim S Yeom J Yeom S Seok Kim B The role of periplasmic antioxidant enzymes (superoxide dismutase and thiol peroxidase) of the Shiga toxin-producing *Escherichia coli* O157:H7 in the formation of biofilms. Proteomics (2006) 6(23):6181–93. 10.1002/pmic.200600320 17133368

[86] Lawrenz MB Miller VL. Comparative analysis of the regulation of *rovA* from the pathogenic yersiniae. J Bacteriol (2007) 189(16):5963–75. 10.1128/JB.00528-07 17573476 PMC1952055

[87] Revell PA Miller VL. A chromosomally encoded regulator is required for expression of the *Yersinia enterocolitica inv* gene and for virulence. Mol Microbiol (2000) 35(3):677–85. 10.1046/j.1365-2958.2000.01740.x 10672189

[88] Shimada T Yamamoto K Ishihama A. Involvement of the leucine response transcription factor LeuO in regulation of the genes for sulfa drug efflux. J Bacteriol (2009) 191(14):4562–71. 10.1128/JB.00108-09 19429622 PMC2704711

[89] Shimada T Bridier A Briandet R Ishihama A. Novel roles of LeuO in transcription regulation of *E. coli* genome: antagonistic interplay with the universal silencer H-NS. Mol Microbiol (2011) 82(2):378–97. 10.1111/j.1365-2958.2011.07818.x 21883529

[90] Turnbull AL Kim W Surette MG. Transcriptional regulation of *sdiA* by cAMP-receptor protein, LeuO, and environmental signals in *Salmonella enterica* serovar Typhimurium. Can J Microbiol (2012) 58(1):10–22. 10.1139/W11-101 22149171

[91] Michael B Smith JN Swift S Heffron F Ahmer BM. SdiA of *Salmonella enterica* is a LuxR homolog that detects mixed microbial communities. J Bacteriol (2001) 183(19):5733–42. 10.1128/JB.183.19.5733-5742.2001 11544237 PMC95466

[92] Smith JN Ahmer BM. Detection of other microbial species by *Salmonella*: expression of the SdiA regulon. J Bacteriol (2003) 185(4):1357–66. 10.1128/JB.185.4.1357-1366.2003 12562806 PMC142872

[93] Ahmer BM Smith JN Dyszel JL Lindsay A. Methods in cell-to-cell signaling in *Salmonella*. Methods Mol Biol (2007) 394:307–22. 10.1007/978-1-59745-512-1_15 18363242

[94] Gorshkov V Petrova O Gogoleva N Gogolev Y. Cell-to-cell communication in the populations of enterobacterium *Erwinia carotovora* ssp. atroseptica SCRI1043 during adaptation to stress conditions. FEMS Immunol Med Microbiol (2010) 59(3):378–85. 10.1111/j.1574-695X.2010.00684.x 20528924

[95] Mei GY Yan XX Turak A Luo ZQ Zhang LQ. AidH, an alpha/beta-hydrolase fold family member from an *Ochrobactrum* sp. strain, is a novel *N*-acylhomoserine lactonase. Appl Environ Microbiol (2010) 76(15):4933–42. 10.1128/AEM.00477-10 20525860 PMC2916461

[96] Weeks JN Galindo CL Drake KL Adams GL Garner HR Ficht TA. *Brucella melitensis* VjbR and C12-HSL regulons: contributions of the *N*-dodecanoyl homoserine lactone signaling molecule and LuxR homologue VjbR to gene expression. BMC Microbiol (2010) 10:167. 10.1186/1471-2180-10-167 20529360 PMC2898763

[97] Guadarrama C Medrano-López A Oropeza R Hernández-Lucas I Calva E. The *Salmonella enterica* Serovar Typhi LeuO global regulator forms tetramers: residues involved in oligomerization, DNA binding and transcriptional regulation. J Bacteriol (2014) 196(12):2143–54. 10.1128/JB.01484-14 24659766 PMC4054188

[98] VanBogelen RA Olson ER Wanner BL Neidhardt FC. Global analysis of proteins synthesized during phosphorus restriction in *Escherichia coli*. J Bacteriol (1996) 178(15):4344–66. 8755861 10.1128/jb.178.15.4344-4366.1996 PMC178200

[99] Fang M Majumder A Tsai KJ Wu HY. ppGpp-dependent *leuO* expression in bacteria under stress. Biochem Biophys Res Commun (2000) 276(1):64–70. 10.1006/bbrc.2000.3440 11006083

[100] Majumder A Fang M Tsai KJ Ueguchi C Mizuno T Wu HY. LeuO expression in response to starvation for branched-chain amino acids. J Biol Chem (2001) 276(22):19046–51. 10.1074/jbc.M100945200 11376008

[101] Stratmann T Pul U Wurm R Wagner R Schnetz K. RcsB-BglJ activates the *Escherichia coli leuO* gene, encoding an H-NS antagonist and pleiotropic regulator of virulence determinants. Mol Microbiol (2012) 83(6):1109–23. 10.1111/j.1365-2958.2012.07993.x 22295907

[102] Lawley TD Chan K Thompson LJ Kim CC Govoni GR Monack DM. Genome-wide screen for *Salmonella* genes required for long-term systemic infection of the mouse. PLoS Pathog (2006) 2(2):e11. 10.1371/journal.ppat.0020011 16518469 PMC1383486

[103] Rodríguez-Morales O Fernández-Mora M Hernández-Lucas I Vázquez A Puente JL Calva E. *Salmonella enterica* serovar Typhimurium *ompS1* and *ompS2* mutants are attenuated for virulence in mice. Infect Immun (2006) 74(2):1398–402. 10.1128/IAI.74.2.1398-1402.2006 16428792 PMC1360296

[104] Moorthy S Watnick PI. Identification of novel stage-specific genetic requirements through whole genome transcription profiling of *Vibrio cholerae* biofilm development. Mol Microbiol (2005) 57(6):1623–35. 10.1111/j.1365-2958.2005.04797.x 16135229 PMC2600799

[105] Chatfield SN Dorman CJ Hayward C Dougan G. Role of OmpR-dependent genes in *Salmonella typhimurium* virulence: mutants deficient in both *ompC* and *ompF* are attenuated *in vivo*. Infect Immun (1991) 59(1):449–52. 1846127 10.1128/iai.59.1.449-452.1991 PMC257763

[106] Gil F Ipinza F Fuentes J Fumeron R Villarreal JM Aspee A The *ompW* (porin) gene mediates methyl viologen (paraquat) efflux in *Salmonella enterica* serovar Typhimurium. Res Microbiol (2007) 158(6):529–36. 10.1016/j.resmic.2007.05.004 17618087

[107] Moreno-Eutimio MA Tenorio-Calvo A Pastelín-Palacios R Pérez-Shibayama C Gil-Cruz C López-Santiago R *Salmonella* Typhi OmpS1 and OmpS2 porins are potent protective immunogens with adjuvant properties. Immunology (2013) 139(4):459–71. 10.1111/imm.12093 23432484 PMC3719063

[108] Espinosa E Casadesús J. Regulation of *Salmonella enterica* pathogenicity island 1 (SPI-1) by the LysR-type regulator LeuO. Mol Microbiol (2014) 91(6):1057–69. 10.1111/mmi.12500 24354910

[109] Fahlen TF Mathur N Jones BD. Identification and characterization of mutants with increased expression of *hilA*, the invasion gene transcriptional activator of *Salmonella typhimurium*. FEMS Immunol Med Microbiol (2000) 28(1):25–35. 10.1111/j.1574-695X.2000.tb01453.x 10767604

[110] Lostroh CP Lee CA. The *Salmonella* pathogenicity island-1 type III secretion system. Microbes Infect (2001) 3(14–15):1281–91. 10.1016/S1286-4579(01)01488-5 11755416

[111] Sturm A Heinemann M Arnoldini M Benecke A Ackermann M Benz M The cost of virulence: retarded growth of *Salmonella* Typhimurium cells expressing type III secretion system 1. PLoS Pathog (2011) 7(7):e1002143. 10.1371/journal.ppat.1002143 21829349 PMC3145796

[112] Bustamante VH Calva E. LeuO, a dormant sentinel for SPI-1? Mol Microbiol (2014) 91(6):1054–6. 10.1111/mmi.12514 24405400

[113] Lucchini S Rowley G Goldberg MD Hurd D Harrison M Hinton JC. H-NS mediates the silencing of laterally acquired genes in bacteria. PLoS Pathog (2006) 2(8):e81. 10.1371/journal.ppat.0020081 16933988 PMC1550270

[114] Navarre WW Porwollik S Wang Y McClelland M Rosen H Libby SJ Selective silencing of foreign DNA with low GC content by the H-NS protein in *Salmonella*. Science (2006) 313(5784):236–8. 10.1126/science.1128794 16763111

[115] Dorman CJ H-NS, the genome sentinel. Nat Rev Microbiol (2007) 5(2):157–61 10.1038/nrmicro1598 17191074

